# Chronic Kidney Disease of Unknown Etiology (CKDu) in Sri Lanka: Hematological Changes and Pro-Inflammation Suggest Likely Predictors of Advance Disease, as Renal Outcomes Show Prevalent Normoalbuminuria

**DOI:** 10.3390/diseases10010002

**Published:** 2021-12-24

**Authors:** S. H. Nandana P. Gunawickrama, K. Imesha G. Hewavitharana, P. G. Chandra L. Nanayakkara, K. B. Suneetha Gunawickrama

**Affiliations:** 1 Institute for Combinatorial Advance Research and Education, General Sir John Kotelawala Defence University, Ratmalana 10390, Sri Lanka; kanchana.hewavitharana@yahoo.com; 2 Department of Anatomy, Faculty of Medicine, University of Ruhuna, Galle 80000, Sri Lanka; pgclnana@med.ruh.ac.lk; 3 Department of Zoology, Faculty of Science, University of Ruhuna, Matara 81000, Sri Lanka; suneetha@zoo.ruh.ac.lk

**Keywords:** chronic kidney disease of unknown etiology, chronic kidney disease development, hematology, nonproteinuria, monocyte chemoattractant protein-1, interleukin-6, basophils, lymphocytes, neutrophil to lymphocyte ratio, mean platelet volume

## Abstract

CKDu needs to be characterized in fundamental areas to improve etiological understanding and disease management. In a cross-sectional study, blood cell profile and plasma inflammatory cytokines were followed by automated analysis and sandwich ELISA, respectively. Disease development stages and proteinuria were ascertained by eGFR and UACR. Comparison among control and stages (ANOVA/Dunnett’s MRT) revealed time-specific changes (*p* < 0.05), including decreased erythrocytes (G5) and hematocrit (G5), and increased MCHC (G3b, G4), MCV (G5), and MCH (G5). CKDu decreased (*p* < 0.05) lymphocytes (G3b, G4, G5), monocytes (G3b), MPV (G3b, G4, G5), and plateletcrit (G3b, G4), and increased basophils (G3a, G3b, G4), N/L (G4) and PLR (G4–G5). MCHC and aforesaid leukocyte variables were in correlation (rho > ±0.03, *p* < 0.05, Pearson’s test) with disease development. MCP-1 and IL-6 spiked (*p* > 0.05) at G3b. Multivariate analyses confirmed that MCP-1, lymphocytes, and BMI were related to renal dysfunction, pointing to inflammation, compromised immunity, and muscle wasting as CKDu effects. Nonproteinuric CKDu was prevalent (23.2–35.6% of total CKDu) with (*p* < 0.05) elevated basophils (G3a), N/L (G4), and depleted lymphocytes (G4). In both forms, G1–G2 were unaffected, and the earliest change was G3a basophils. Results suggest that MCP-1, lymphocyte count, N/L, and PLR may verify the stage and predict impending ESRD in advance proteinuric CKDu.

## 1. Introduction

Chronic kidney disease of unknown etiology (CKDu) in Sri Lanka is endemic nephropathy in the country’s North Central and Uva provinces [1]. CKDu is distinct as it lacks etiologic association with CKD initiation risk factors such as diabetes mellitus and hypertension [2]. Nevertheless, it obeys diagnostic criteria based on poor renal outcomes in terms of estimated glomerular filtration rate (eGFR) and urine albumin to creatinine ratio (UACR) [3].

Early CKDu is marked by inflammatory infiltration into tubulointerstitium, tubulitis, fibrosis and glomerular scarring [4] that progresses to end-stage renal disease (ESRD) possibly via chronic ischemia attributed to interstitial damage [5]. Histopathology and tissue damage points to an inflammatory component in disease progression. CKD effects such as anemia [6], compromised immunity [7] and inflammation [8] manifest in blood. In this context, it is important that the CKDu endemic in Sri Lanka be characterized for pertaining changes with time-points of emergence during disease progression. Whole blood cellular and biochemical changes are easily detectable so that such comprehension will improve diagnosis and prediction capacities while bolstering etiology-insight. For this purpose, the study followed changes in the blood, immune and inflammatory cell profile and cytokine levels during CKDu progression from good renal outcomes (control) to end-stage renal disease (ESRD). It also examined the key etiologic dichotomy of CKD, proteinuria, and nonproteinuria (normoalbuminuria) in endemic CKDu of Sri Lanka in a novel effort. Clarity, in this regard, is important as albuminuria is a key marker in the screening and diagnosis of renal disease.

## 2. Materials and Methods

**Scheme 1:**
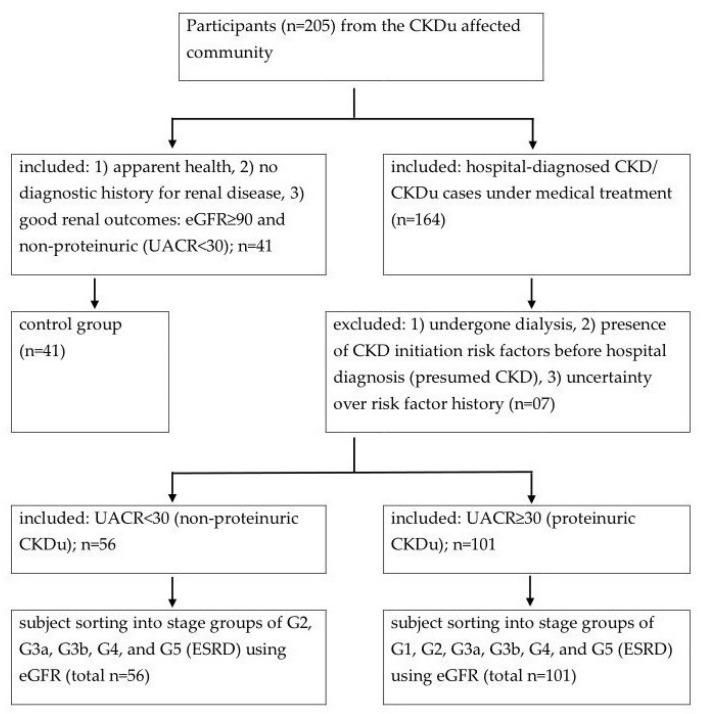
Subject sorting into study-groups at CKDu endemic Girandurukotte/Mahiyanganaya area, CKD: chronic kidney disease, eGFR: estimated glomerular filtration rate (mL/min/1.73 m^2^), UACR: urine albumin to creatinine ratio (mg/g), G1–G5: CKD development stages, ESRD: end-stage renal disease, Study relied on its own spot eGFR and UACR determinations.

A cross-sectional study was conducted in the CKDu endemic Girandurukotte/Mahiyanganaya area (GK/MH) in the Uva province of Sri Lanka. Following informed consent, volunteers who were either presumably healthy or under medical attention for diagnosed CKD/CKDu participated (*n* > 205) in the study (Scheme 1). Participants who had CKD initiation risk factors (IRF) [9] before initial diagnosis with poor renal outcomes were considered to be CKD and excluded. IRF were ascertained by subject interview, and included diabetes, hypertension, systemic infections, urinary tract infections, urinary stones, lower urinary tract obstructions, and family history of renal disease. Peripheral whole blood (3–5 mL), spot urine, and demographic information were collected from the rest under medical and ethical supervision. Sample collection was carried out in the 2017–2018 period of the study.

### 2.1. Initial Sample Processing

Aliquots of EDTA-whole blood were dispatched on ice for same-day cell counts with an automated hematology analyzer (HumaCount 5 L, Wiesbaden) at a commercial lab. Plasma and serum were obtained by centrifugation (3000 rpm × 10 min at RT) of EDTA-whole blood and whole blood allowed to clot (30 min at RT), respectively. Supernatants were stored on dry ice for transportation before final storage at −80 °C in a freezer at the laboratory.

### 2.2. Kidney Dysfunction Markers

Serum creatinine (sCr, mg/dL), urine albumin (mg/L), and urine creatinine (mg/dL) were measured by enzymatic colorimetric, immunoturbidimetric, and Jaffe assays, respectively (Mindray BS-200 clinical chemistry analyzer, Shenzhen, China). Serum Cystatin C (sCysC, mg/L) was measured (QR100- Heales specific protein analyzer, Shenzhen, China) by latex-enhanced turbidimetric assay. All assays adhered to the respective quality control measures and protocols of the manufacturer.

Estimated glomerular filtration rate (eGFR, mL/min/1.73 m^2^) was calculated by chronic kidney disease epidemiology collaboration (CKD-EPI) equation using sCr [10]. In addition, eGFR was also estimated by the equations, abbreviated modification of diet in renal disease (MDRD) by sCr, CKD-EPI by sCysC, and CKD-EPI by sCr and sCysC as described elsewhere [10,11]. Urine albumin to creatinine ratio (UACR, mg/g) was estimated using urine albumin (mg/L) and urine creatinine (mg/dL).

### 2.3. Subject Sorting into Study Groups

The eGFR (CKD-EPI, sCr) and UACR were used in sorting of subjects into control and CKD stages in compliance with the accepted protocol in studies [3,12]. Briefly, IRF-verified CKDu subjects were provisionally sorted by eGFR into CKD stage groups as G1 ≥ 90, G2; 89–60, G3a: 59–45, G3b: 44–30, G4: 29–15, and G5 (end-stage renal disease/ESRD) < 15. Subjects at G1–G5 stages were further classified either as proteinuric (UACR ≥ 30), or nonproteinuric (normoalbuminuric, UACR < 30) as described before [13]. The control group was constituted to have eGFR ≥ 90 and UACR < 30 and thus to have good renal health.

### 2.4. Cytokine Measurements

A range of six pro-inflammatory mediators in plasma were determined by quantitative sandwich-enzyme linked immunosorbent assay (ELISA). ThermoFisher Scientific^®^ (Waltham, MA, USA) test kits; BMS243-2, KAC1261, BMS228, BMS2034 and BMS281 were used for interleukin-1 alpha (IL-1α), interleukin-6 (IL-6), interferon-gamma (IFN-γ), tumor necrosis factor-alpha (TNF-α) and monocyte chemoattractant protein-1 (MCP-1) respectively.

### 2.5. Statistical Analyses

Each continuous dependent variable was tested for statistical significance among control and disease-development-stage groups and for linear association with disease progression using one-way ANOVA followed by Dunnett’s multiple range test and Pearson’s correlation method respectively. Statistical significance (*p* < 0.05) in group comparison was a prerequisite for any variable to be tested for correlation. Multivariate analyses were performed to ascertain non-linear relationships among variables that showed a minimum correlation at or beyond *r* = ±0.03 and *p* < 0.05 (Pearson’s test) with any of the kidney dysfunction markers of the study. Principal component analysis (PCA) by correlation-based matrix was performed subsequently to identify clustered variables. It was followed by the development of a cluster dendrogram for added data visualization, involving complete linkage and correlation coefficient distance. The progressive variable selection was expected to keep stringency and highlight the most dependable marker candidates at the end. Statistical procedures and plotting were conducted on Minitab 17 statistical software.

### 2.6. Assessment of Nonproteinuria

Log_10_ transformed eGFR and UACR were plotted to visualize nonproteinuria and proteinuria conceptually and in relation to the disease development. Data from the present GK/MH study were projected with data from CKDu endemic Padaviya (PDV) in North Central Province for comparison. PDV study was ratified (RP/2015/04) by the ethical review committee mentioned before (Section 2) and had identical sampling, marker analyses, and subject sorting procedures to the GK/MH study.

## 3. Results

Recruited proteinuric CKDu subjects comprised 52% males and 48% females and were within a 19–86 years age range in GK/MH. In comparison to control (*n* = 41), the peak CKDu prevalence occurred between 40–69 years of age as stages G3a, G3b, and G4 spanned the highest incidence (data not shown). The affected comprised of paddy farmers (44%), laborers (5%) and unemployed (35%) in a total of 157 CKDu cases. In PDV, CKDu subjects (*n* = 176) were predominantly comprised of paddy farmers (89.2%) and government servants (10.6%) and were within a 36–77 years age range. Endemic control had 14 subjects who were scarce.

**Figure 1:**
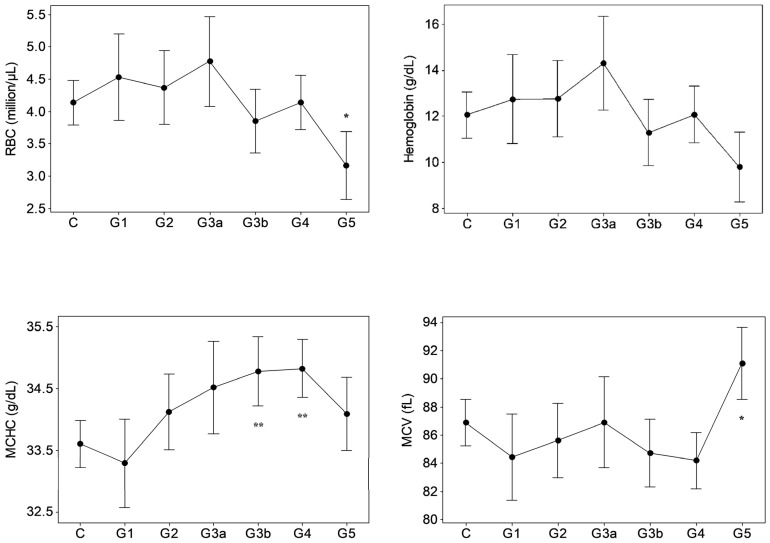

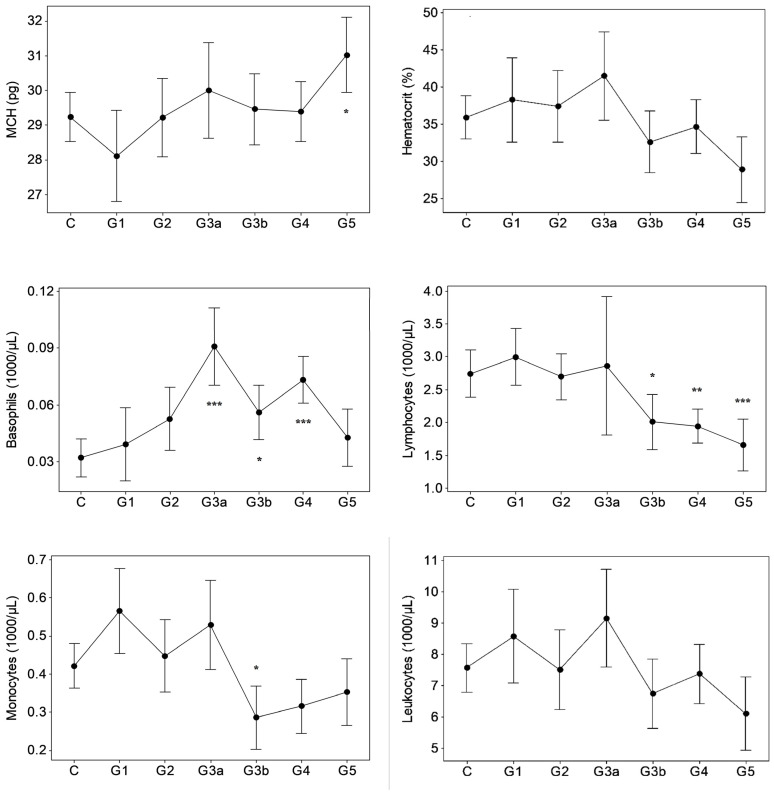

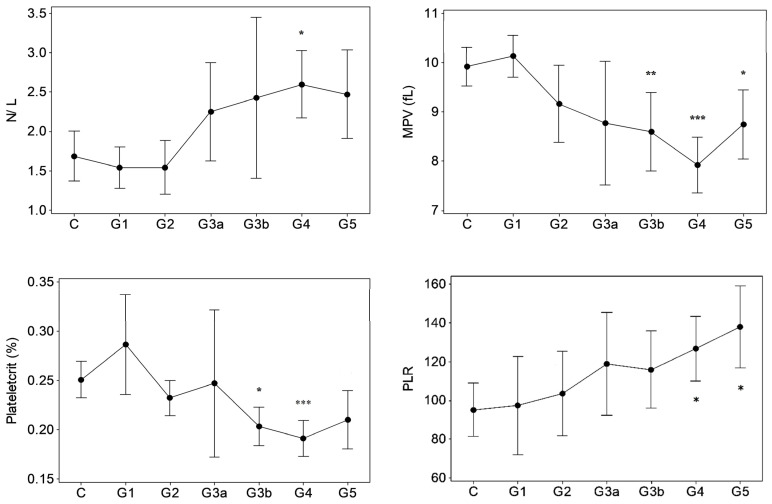
Hematological variations in proteinuric CKDu progression. *n* = 38–41, 11, 15, 10, 18–20, 26–27, 16–18 in C (control) and G1–G5 (CKD staging) respectively. Data presented as mean and 95% confidence interval. * *p* < 0.05, ** *p* < 0.01, *** *p* < 0.001 compared to control (one way ANOVA, Dunnett’s MRT). Plots with no asterisk: *p* < 0.05 by ANOVA and *p* > 0.05 by Dunnett’s MRT. RBC: red blood cells, MCMC: mean corpuscular hemoglobin concentration, MCV: mean corpuscular volume, MCH: mean corpuscular hemoglobin, N/L: neutrophil to lymphocyte ratio, MPV: mean platelet volume, PLR: platelet to lymphocyte ratio.

**Figure 2:**
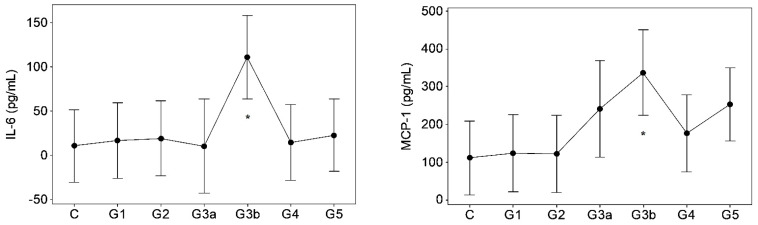
Inflammatory mediators in proteinuric CKDu progression. *n* = 12, 11, 11, 7, 9, 11, 12 in C (control) and G1–G5 (CKD staging) respectively (see Figure 1 foot note for statistical methods and symbols). IL-6: interleukin-6, MCP-1: monocyte chemoattractant protein (plasma IL-1, TNF-α, and INF-γ were *p* > 0.05, data not shown).

Results of GK/MH collectively showed significant changes (*p* < 0.05) in certain blood cell counts and indices (Figure 1) and plasma pro-inflammatory cytokine levels (Figure 2). The changes emerged at stage G3a or beyond in the disease development process with notably unaffected G1 and G2. Compared to control, the red blood cell (RBC) count decreased (*p* < 0.05) at ESRD. Significant increases were seen in the mean corpuscular hemoglobin concentration (MCHC) at G3b and G4, the mean corpuscular volume (MCV) at G5, and in the mean corpuscular hemoglobin (MCH) at G5. MCHC changes associated progressively deteriorating renal function in terms of eGFR (*r*: −0.343, *p* < 0.001, Table 1). Developing CKDu reduced (*p* < 0.05) total lymphocytes and the mean platelet volume (MPV). Lymphocyte depletion was significant from stage G3b (*p* < 0.05) and further aggravated via G4 (*p* < 0.01) to ESRD (*p* < 0.001). Transient increases (*p* < 0.05) were seen in the basophil numbers at G3a, G3b and G4 and the neutrophil to lymphocyte ratio (N/L) at G4, with ESRD, however, unaffected by both variables. G3b monocyte count and G3b–G4 plateletcrit (PCT) were lower (*p* < 0.05) as well. Platelet to lymphocyte ratio (PLR) was elevated from G4 to ESRD. Again, altered basophil, lymphocyte and monocyte numbers, and N/L, MPV and PCT as well as PLR showed linear associations with declining kidney function by eGFR (*r* > ±0.3, *p* < 0.05). Pro-inflammatory cytokines, IL-6 and MCP-1 developed solitary spikes at G3b (*p* > 0.05, Figure 2) in plasma with no correlation with any of the five kidney dysfunction markers deployed.

**Figure 3:**
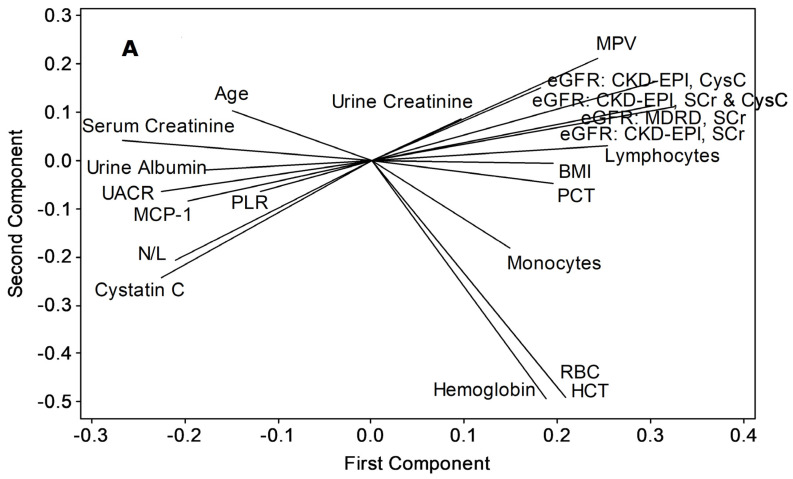

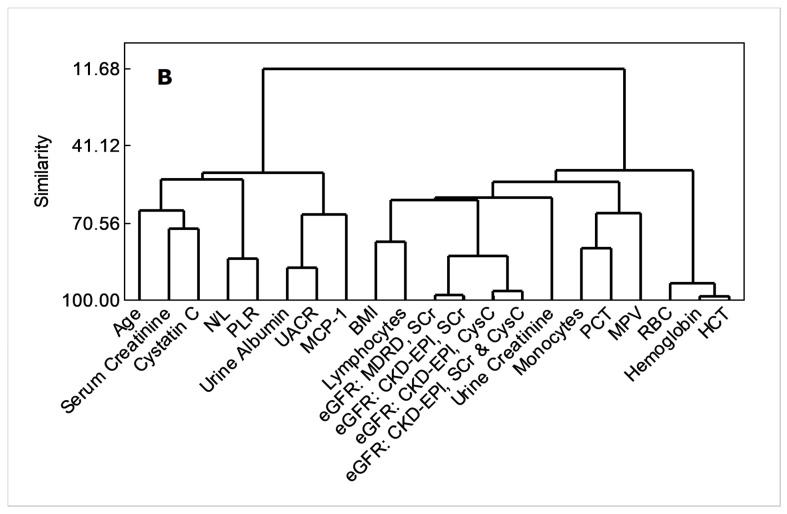
Applicability of MCP-1, total lymphocytes and N/L as advance-stage markers of proteinuric CKDu progression, (**A**) principal component analysis/ loading plot, (**B**) cluster dendrogram. Variables were included if a correlation coefficient beyond ±0.3 at *p* < 0.05 is shown with any of UACR or eGFR variants (Pearson’s test, *n* = 12 control + 61 CKDu cases from all stages). HCT (%): hematocrit (see Figure 1 and Figure 2, and Table 1 for acronym definitions and units).

Both principal component analyses and cluster dendrogram (Figure 3) projected UACR as having the closest non-linear relations with MCP-1 (similarity, 67.21%), while eGFR variants were related closely to body mass index (BMI, similarity, 61.81%) and lymphocyte count (similarity, 61.81%).

**Figure 4:**
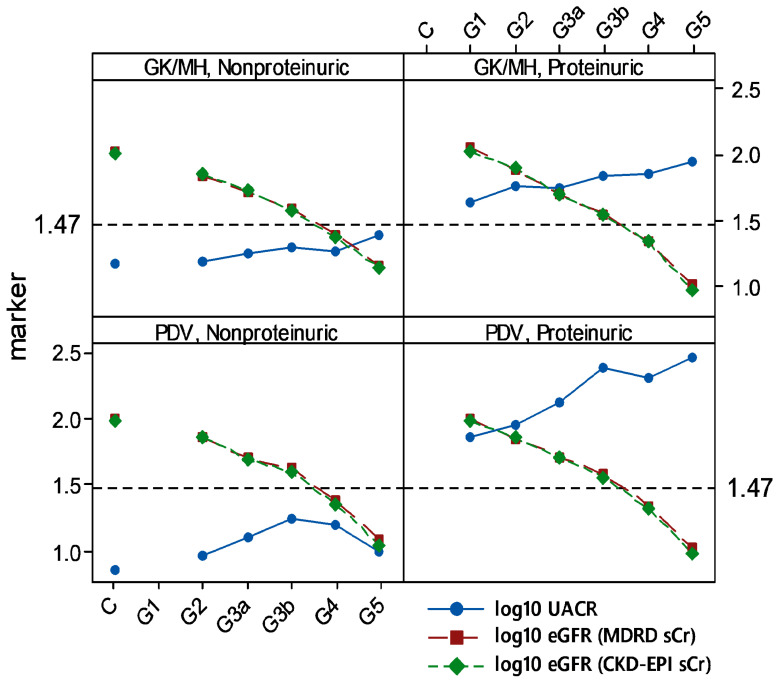
Normoalbuminuria among endemic CKDu cases of Sri Lanka. Means of log10 transformed data are shown. Dashed horizontal line at 1.47 represents log_10_ of UACR = 30 mg/g, low limit of proteinuria. *n* (in X-axis order; control, G1–G5): Girandurukotte/ Mahiyanganaya (GK/MH) = 40, none, 25, 11, 6, 13, 1 of nonproteinuria, and none, 11, 15, 10, 20, 27, 18 of proteinuria; Padaviya (PDV) = 14, none, 21, 7, 4, 8, 1 of nonproteinuria, and none, 8, 16, 14, 22, 42, 33 of proteinuria.

Nonproteinuria prevailed among 23.29% and 35.66% of total CKDu subjects recruited from endemic GK/MH and PDV, respectively. Among proteinuric CKDu subjects in both areas, kidney dysfunction (by eGFR < 90) and proteinuria (by UACR ≥ 30) emerged at G1 (Figure 4). It was further evident when nonproteinuric control was considered as the reference. On the contrary, in nonproteinuria of GK/MH and PDV, stages G2, G3a, G3b, and G4 were persistently normoalbuminuric (UACR < 30).

Nonproteinuric CKDu subjects in GK/MH (Table 2) showed significant increases in basophil level at G3a (*p* < 0.001) and N/L at G4 (*p* < 0.01), amid lymphocyte depletion at G4 (*p* < 0.05).

## 4. Discussion

CKD and CKDu cases coexist in CKDu endemic areas of Sri Lanka [1,14,15]. Since CKD ensues its initiation risk factors [9], a lack of such history in hospital-diagnosed CKD/CKDu patients was considered as proof of a CKDu subject. The present study involved volunteers from all levels of the disease development and the healthy, who were sorted on eGFR and verified with UACR. This approach permitted the disease progression to be followed from good renal outcomes (normoalbuminuric control with eGFR ≥ 90) to CKD stages G1 through G5 (ESRD) at one time-point in a cross-sectional design. Essentially, eGFR, UACR and stage classification established kidney dysfunction or the CKDu progression axis that allowed dependent variables to be studied upon it.

Hematology variations (*p* < 0.05) in proteinuric CKDu development commenced with G3a basophil count (Figure 1) and correlated with progressive kidney dysfunction (Table 1), suggesting that renal failure could be causal. Such variables in concert may constitute a predictor of advance CKDu stages subjected to reproducibility and early detection. Candidates are G3a basophils and G3b MCHC, lymphocytes, monocytes, MPV and PCT, and G4 N/L. In nonproteiuria, G3a Basophils followed by G4 lymphocytes and N/L may be considered.

A common cellular trend was that an increase in MCHC at G3b and G4 was followed by decreased RBC at ESRD (*p* < 0.05). Further, at ESRD, total hemoglobin (HB) and hematocrit (HCT) apparently dropped (intergroup variation; *p* < 0.05 by ANOVA, *p* > 0.05 by Dunnett’s MRT) as well, whereas MCV and MCH had significantly risen (*p* < 0.05). Results conform anemia, which is a CKD outcome [6,16]. Plausible mechanisms include iron and erythropoietin deficiency and shortened erythrocyte lifespan that emerge as complications [17]. Temporal trends show that RBC, HB and HCT declines manifest as late as ESRD/CKDu, with no statistical association to dysfunction markers. Red cell distribution width (RDW) is a measure of red cell size variation. CKD patients have high RDW levels [16] which perpetuate across CKD stages [18]. Unaffected RDW in CKDu remains intriguing in the present study.

With the progression of CKDu, neutrophil and eosinophil counts did not change (*p* > 0.05), but basophil numbers remained consistently high at G3a, G3b and G4, and correlated to CKDu progression. Basophils are the key cellular mediators of type I hypersensitivity reactions, so, arguably, the cells may have an inflammatory role in CKDu progression. Elevated N/L has also been postulated as an indicator of inflammation that associates poor renal outcomes [19], particularly at advance CKD stages [20]. The prognostic value of N/L, particularly at CKD stage 4, was appreciated [21]. As results show, N/L increased at G4 in correlation with CKDu progression in the GK/MH area and could be linked to unaltered neutrophils and decreased lymphocyte counts at the stage.

Significantly declined peripheral blood lymphocyte count at G3b, G4, and ESRD was in correlation with kidney dysfunction. Lymphocyte depletion is consistent with immunodeficiency at CKD advance stages [7,22,23,24]. Transiently increased plasma MCP-1 at G3b (Figure 2) and depleted peripheral blood monocytes (G3b) in correlation with kidney dysfunction could be mechanistically related in the possible event of monocyte infiltration into renal tissue [25]. Increased plasma MCP-I should result from the upregulation of expression rather than from poor renal clearance during the disease [26]. MCP-1 elevation has been reported in association with decreased eGFR in CKD [27]. The plasma IL-6 spike in CKDu subjects at G3b is notable as it is a multifunctional cytokine that has modulatory roles on different but overlapping facets of CKD, such as anemia [28], acute phase response, monocyte differentiation into macrophages, and T cell responses [29]. Increased plasma IL-6 is common in CKD, and chronic inflammation, poor renal clearance, and emerging stress factors may be the inducers.

Advance stages of chronic renal failure affect platelet integrity and function. The common endpoints are platelet abnormalities [30] and uremic platelet dysfunction [31]. Among CKD subjects, a lower MPV positively relating to eGFR [32,33] and reduced platelet reactivities [33] were reported. Significant reductions in MPV and PCT from G3b in the present study may show an onset of hemorrhagic tendencies quite earlier than ESRD in CKDu development, suggesting a predictor value of the variables concerned. Increased PLR has been evaluated for its inflammatory role and appearance in pre-dialysis and ESRD stages [34]. In GK/MH CKDu subjects from G4 onwards, unaffected (*p* > 0.05) platelet count may be a reason for high PLR amid concomitantly depleted lymphocytes.

Consideration of PCA and dendrogram (similarity > 60%) confirms that MCP-1, total lymphocytes, and BMI as the individual parameters that closely follow progressive kidney dysfunction in endemic CKDu (Figure 3). BMI showed a significant (*p* < 0.05) intergroup variation by ANOVA without confirmation (*p* > 0.05) from Dunnett’s MRT (data not shown). This, together with N/L and PLR responses (Figure 1, Table 1), which were related with a similarity of 83.9% (Figure 3) indicate that continued inflammation, compromised immunity, and muscle wasting ensue renal health deterioration into ESRD.

The division of proteinuria and nonproteinuria reflects two pathways, which converge in ESRD via tubular and interstitial damage [35]. The former involves reabsorption of albumin from the glomerular filtrate. It results in local generation of reactive oxygen species (ROS) and activation of both immune and inflammatory agents in a sustained manner [35]. The nonproteinuric pathway appears to rely on factors such as hyperfiltration at nephrons [35], which is linked to a low nephron number at birth. However, whether such risk factors may describe CKDu remains to be uncertain. Literature review convinced authors that nonproteinuria in CKDu of Sri Lanka has neither been investigated nor reviewed with focus. Intriguingly, presence of normoalbuminuria (23–35% among total CKDu) in the study is suggestive of two unknown etiologies in CKDu development in the country (Figure 4). Concept plots derived were consistent between the endemic areas studied. It provides proof that endemic CKDu at GK/MH and PDV comprises both proteinuirc and nonproteinuric renal disease. This trend is likely to be common in entire geographical area of CKDu endemicity as well. Nonproteinuric (UACR < 30) G1 subjects were nonexistent in data since G1 represents eGFR ≥ 90, and the criteria together classify the cases among good renal outcomes. One subject each was identified (*n* = 1) at nonproteinuria/ESRD/CKDu in GK/MH (*n* = 56) and PDV (*n* = 41). The dearth of cases at ESRD compared to proteinuric/ESRD (*n* = 18 of GK/MH, and *n* = 33 of PDV) points to the likelihood that UACR may increase towards advance stages in nonproteinuria. Question about whether the nonproteinuric CKD may develop proteinuria at or before ESRD had been raised before [14].

Nonproteinuria among CKDu cases should be compromising community screening and initial diagnosis of the chronic renal disease under current criteria [36] as UACR ≥ 30 only points to proteinuric CKDu. The practice overlooks normolbuminuric CKDu and CKD. The issue may be circumvented by relying more on serial measurement of eGFR and adopting a marker that has been validated for early renal damage. The notion that CKDu in Sri Lanka typically associates minimal proteinuria at early stages [37] may be arguable since the presence of both CKDu categories may underestimate true albuminuria in group statistics. Results of the present study showed albuminuria amongst the majority of CKDu cases (64–76%), which spanned from G1 to G5 (ESRD) in both endemic areas studied.

Basophil, lymphocyte, and N/L variations in nonproteinuria, and their associations with kidney dysfunction (Table 2), appear to be corresponding to proteinuria, considering the time-points of emergence during CKDu progression. However, unchanged (*p* > 0.05) MCP-1, IL-6, and monocyte/macrophage counts in peripheral blood over the disease progression point only to a moderate inflammation compared to the proteinuric disease development. Similarly, unaffected (*p* > 0.05) erythrocyte/hemoglobin and platelet variables in nonproteinuria suggest a lesser impact of disease development as compared to proteinuric disease. Scarcity of nonproteinuria at ESRD posed the constraint in verifying associated blood responses, such as anemia, at the final stage.

## 5. Conclusions

Notably, about a quarter of CKDu-affected people in endemic areas in Sri Lanka could be normoalbuminuric, which remained unaffected despite disease progression. This novel reporting also indicates that albuminuria-based community screening and diagnosis may overlook a sizable fraction with the renal disease and emphasizes the need for an early renal dysfunction marker to complement eGFR and offset the constraint. Changes in peripheral blood cell counts, indices, and pro-inflammatory cytokines emerged half-way through disease development and did not qualify to be early markers. The changes, however, were attributes of the advance disease. Analyses showed that the MCP-1 spike at G3b, lymphocyte depletion spanning G3b–G5, and elevated N/L and PLR at G4, and G4–G5, respectively, would possibly verify the stage and predict ESRD in proteinuric CKDu. Augmented G3a–G5 basophils and declined G3b–G5 MPV were persistent in the advance disease and in association with progressive renal dysfunction to be further candidates as stage markers and predictors. Reproducibility efforts, mechanistic elucidation, and markers in concert should be further directions.

## Figures and Tables

**Table 1:** Hematological changes follow renal dysfunction in proteinuric CKDu.

	eGFR (MDRD, sCr) mL/min/1.73 m^2^	eGFR (CKD-EPI, sCr) mL/min/1.73 m^2^	UACR mg/g
MCHC (g/dL)	−0.303 *	−0.343 *	
Basophils (10^3^ µL)		−0.308 *	
Lymphocytes (10^3^ µL)	0.382 *	0.411 *	
Monocytes (10^3^ µL)	0.318 *	0.304 *	
N/L		−0.331 *	
MPV (fL)	0.461 *	0.480 *	−0.357 *
PCT (%)	0.396 *	0.425 *	
PLR		−0.329 *	

Correlations (*n* = 41 control, and 101 CKDu from all stages of disease development) were considered if r > ±0.3 (Pearson’s test). Values and asterisk represent r and *p* < 0.001 respectively. eGFR: estimated glomerular filtration rate, sCr: serum creatinine, UACR: urine albumin to creatinine ratio, PCT: plateletcrit (see Figure 1 foot note for additional definitions), Voids show no correlation.

**Table 2:** Hematological variations during endemic CKDu progression associated with normoalbuminuria in Girandurukotte/ Mahiyanganaya area.

	Control ^1^	G2 ^1^	G3a ^1^	G3b ^1^	G4 ^1^	G5	eGFR (MDRD, sCr) ^2^	eGFR (CKD-EPI, sCr) ^2^
Basophils (10^3^/µL)	0.03 ± 0.17	0.02 ± 0.02	0.07 ± 0.03 ***	0.05 ± 0.03	0.05 (0.03)	0.08 (NA)	r = −0.30 **	r = −0.36 ***
Lymphocytes (10^3^/µL)	2.76 ± 1.15	2.21 ± 1.24	2.93 ± 0.68	2.73 ± 0.47	1.83 ± 1.15 *	2.38 (NA)	NS	NS
N/L	1.66 ± 0.96	1.60 ± 0.49	1.77 ± 0.60	1.57 ± 0.36	2.69 ± 1.43 **	2.04 (NA)	NS	NS

^1^ intergroup variations were assessed by one way ANOVA and Dunnett’s MRT. Data represent mean ± SD (*n* = 40, 25, 11, 06, and 13 in control and in stages G2–G4 respectively). G5 (*n* = 01) was excluded from analysis (NA). ^2^ correlation with renal dysfunction (Pearson’s test, *n* = 96). * *p* < 0.05, ** *p* < 0.01, and *** *p* < 0.001. NS; *p* > 0.05 (see Figure 1 and Table 1 foot notes for acronym definitions).

## Data Availability

Data utilized in the paper can be made available from the corresponding author for purpose agreed upon.
